# Evaluating effects of normobaric oxygen therapy in acute stroke with MRI-based predictive models

**DOI:** 10.1186/2045-9912-2-5

**Published:** 2012-03-09

**Authors:** Ona Wu, Thomas Benner, Luca Roccatagliata, Mingwang Zhu, Pamela W Schaefer, Alma Gregory Sorensen, Aneesh B Singhal

**Affiliations:** 1 Athinoula A. Martinos Center for Biomedical Imaging, Department of Radiology, Massachusetts General Hospital, 149 13th Street, Charlestown MA 02129, USA; 2 Department of Neurology, Massachusetts General Hospital, 55 Fruit Street, Boston MA 02114, USA; 3 Department of Radiology, Massachusetts General Hospital, 55 Fruit Street, Boston MA 02114, USA

**Keywords:** Stroke, Clinical Trial, Oxygen Therapy, Neuroprotection, MRI multiparametric models

## Abstract

**Background:**

Voxel-based algorithms using acute multiparametric-MRI data have been shown to accurately predict tissue outcome after stroke. We explored the potential of MRI-based predictive algorithms to objectively assess the effects of normobaric oxygen therapy (NBO), an investigational stroke treatment, using data from a pilot study of NBO in acute stroke.

**Methods:**

The pilot study of NBO enrolled 11 patients randomized to NBO administered for 8 hours, and 8 Control patients who received room-air. Serial MRIs were obtained at admission, during gas therapy, post-therapy, and pre-discharge. Diffusion/perfusion MRI data acquired at admission (pre-therapy) was used in generalized linear models to predict the risk of lesion growth at subsequent time points for both treatment scenarios: NBO or Control.

**Results:**

Lesion volume sizes 'during NBO therapy' predicted by Control-models were significantly larger (P = 0.007) than those predicted by NBO models, suggesting that ischemic lesion growth is attenuated during NBO treatment. No significant difference was found between the predicted lesion volumes at later time-points. NBO-treated patients, despite showing larger lesion volumes on Control-models than NBO-models, tended to have reduced lesion growth.

**Conclusions:**

This study shows that NBO has therapeutic potential in acute ischemic stroke, and demonstrates the feasibility of using MRI-based algorithms to evaluate novel treatments in early-phase clinical trials.

## Background

MRI voxel-based predictive algorithms based on admission MRI datasets including diffusion-weighted imaging (DWI) and perfusion-weighted imaging (PWI), have been shown to accurately predict tissue outcomes in acute stroke patients [1,2] and animal stroke models [3-6]. In the acute stroke setting, these models can be used to predict the patient's response to various treatment options, enable better treatment selection, and thereby improve patient outcome. Furthermore, MRI-based algorithms have potential to be used in early-phase clinical trials to assess the efficacy and toxicity of investigational therapies by providing an estimate of tissue likely to infarct *without *intervention, and comparing this predicted outcome to the *actual *tissue status observed post-treatment. Such algorithms have been successfully used to demonstrate therapeutic efficacy of recombinant tissue plasminogen activator (rt-PA) in animal [3] and human [7] studies. In these two studies, the group given rt-PA had smaller actual lesion volumes than was predicted using natural history models.

In this study, we extend our prior research by developing models under *treatment *and *control *conditions in order to predict tissue outcome after normobaric oxygen therapy (NBO), a promising investigational acute stroke treatment [8-17]. We analyzed data from a prospective randomized pilot study of normobaric oxygen therapy (NBO) [18-20]. In the pilot study [18], NBO transiently improved clinical deficits and reduced DWI lesions in stroke patients with mismatches in lesion volumes on DWI and PWI. These effects were most evident during therapy, suggesting that NBO may slow down the process of ischemic necrosis and hence be a simple strategy to extend the narrow time window for stroke thrombolysis. In the present study we compared predicted tissue outcome on a voxel-wise basis under the two treatment strategies, Room-air and NBO. We postulated that differences between the expected outcomes of these models will objectively validate prior observations and provide insights into NBO's mechanisms.

## Methods

### Patients

This study was approved by our Hospital's Human Research Committee (approval number 2001P001176). The NBO pilot study inclusion and exclusion criteria, DWI and PWI imaging methods, and clinical and volumetric results have been published [18-20]. Data from three additional randomized patients (2 treated with NBO, one with room air), obtained after the original report, were incorporated in this analysis. Briefly, patients were eligible if they presented < 12 h after witnessed stroke onset or < 15 h after last seen neurologically intact, and had a > 20% visually-estimated mismatch in acute DWI and PWI lesion volumes. After obtaining informed consent, patients were randomized to room air (Control, n = 8) or NBO (high-flow oxygen delivered via a facemask for 8 hours, n = 11). All patients received MRIs upon admission (pre-treatment), 4 h later (during treatment), 24 h (post-treatment) and before discharge. One patient from each group was excluded due to hyperacute post-ischemic hemorrhage [21]. Another Control patient was excluded due to imaging artifacts. Reperfusion, defined as recanalization of a previously occluded artery on MR-angiography (MRA) or > 50% decrease in mean transit time (MTT) lesion volume, was measured at 4 h and 24 h.

### Image acquisition

DWI was acquired with b-value = 0 s/mm^2 ^and six diffusion-weighted images with b-value = 1000 s/mm^2^. Apparent diffusion coefficient (ADC) maps were calculated as the negative slope of the linear regression fit of the log DWI images versus their b-values. The isotropic DWI map (iDWI) was calculated as the geometric mean of the diffusion-weighted images. The b-value = 0 s/mm^2 ^acquisition was used as the T_2_-weighted image (T_2_WI). Non-uniformity correction was performed on both the iDWI and the T_2_WI data sets [22]. PWI was performed by acquiring dynamic susceptibility contrast-weighted images during the first pass of a bolus of high-magnetic susceptibility contrast agent. Cerebral blood flow (CBF), cerebral blood volume (CBV), mean transit time (MTT), and delay maps (Tmax) maps were calculated using local oscillation-index regularized singular value decomposition with a block-circulant matrix [23] for deconvolving concentration-time curves with an automatically selected arterial input function [24].

### GLM development

Details regarding the development of generalized linear models (GLM) for predicting tissue infarction have been published [7]. Briefly, tissue outcome is modeled as a binary variable, with 1 representing lesioned tissue and 0 non-lesioned tissue. The probability of tissue lesion development (*P*) can then be represented by the logistic function, $\text{P}=\frac{1}{1+{\text{e}}^{-\eta \left(x\right)}}$ (Equation 1), for which η(x) is a linear function of its input parameters, $\eta \left(x\right)=\beta^{\text{T}}x+\alpha$ (Equation 2) and β is the vector of weighting coefficients and α is the bias or intercept term for the GLM. For this study, the input vector, **x**, consisted of the acute T_2_WI, ADC, iDWI, CBF, CBV, MTT and Tmax maps. All images were co-registered using semi-automated image registration software to one another (FLIRT [25]) and to a probabilistic brain atlas [26] (MNI Autoreg [27]), and then normalized with respect to mean values measured in the normal contralateral white matter using previously described techniques [7]. Training regions consisted of tissue lesions delineated by a neuroradiologist as regions of hyperintensities on 4 h or 24 h DWIs or before discharge MRIs (performed approximately 1-week later), and non-lesions defined as remaining ipsilateral hemisphere tissue not demonstrating artifacts on admission MRI. Coefficients of the GLM were calculated using an iterative re-weighted least squares algorithm (R [28]) and then applied to admission MRI to produce lesion risk maps for each patient. For all models, the bootstrapped estimate [29] of the mean of the GLM coefficients were used, with care taken that an equal number of lesion and non-lesion samples were used for each iteration to compensate for imbalanced training data which can lead to models weighted towards predicting the majority class, i.e. non-lesion tissue [30].

Two sets of models were created to predict lesion growth at each of the three subsequent time points: 4 h (during treatment), 24 h (post-treatment) and Discharge. Control-models were trained using all room-air treated patients (n = 6). NBO-models were trained on oxygen-treated patients (n = 10). Lesion risk maps for the NBO-treated cohort were calculated using both types of models. To avoid bias from training and testing on the same data, jack-knifing or leave-one-out approach was used to evaluate the performance of the NBO-models [29]. The coefficients for the models were tested for significance and compared to one another (Z-tests).

### Data analysis

All analyses were performed on co-registered datasets. Manually outlined lesions at 4 h, 24 h and Discharge were used to train the GLMs and to define the *measured lesion volume *(MLV) at each successive time point. *Lesion Change*, defined as the ratio of the MLV at each time point to the admission DWI lesion volume, was compared between the NBO and control groups (unpaired Student *t*-test). The *predicted lesion volume *(PLV) was defined as tissue where GLM-predicted lesion risk was greater than a threshold of 50%. A threshold of 50% was selected since the models were designed to produce the optimal operating point at this cutoff by using an equal number of lesion and non-lesion voxels as part of the bootstrapped training. PLV at each time point using each model (PLV_Control _and PLV_NBO_) were calculated and compared. Regional analysis was performed comparing T_2_WI, ADC, DWI, CBF, CBV, MTT and Tmax in areas where the PLV of both models matched with areas where PLV_Control _were greater than areas of PLV_NBO_. Correlations between the two models were calculated (Pearson's product-moment) at each time point. Correlations were also performed between ratios of PLV_Control _to PLV_NBO _with respect to Lesion Change to predict the expected responsiveness of individual patients to NBO.

Region of interest (ROI) analysis were performed in tissue that was correctly predicted to become lesioned (true positive, TP) by Control-models (TP_Control_) or NBO-models (TP_NBO_), in tissue that was falsely predicted to become lesioned (false positive, FP) using the Control-model (FP_Control_) or the NBO-model (FP_NBO_), in tissue correctly predicted to not become a lesion (true negative, TN) for Control-models (TN_Control_) or NBO-models (TN_NBO_), and in tissue incorrectly predicted to not become lesioned (false negative, FN) for Control-models (FN_Control_) or NBO-models (FN_NBO_). Mean predicted lesion risk in each region was calculated and compared (ANOVA with post-hoc Tukey HSD test) for each model. All statistical comparisons used one-sided Wilcoxon-tests, unless otherwise noted. P < 0.05 was considered significant for all analyses, which were limited to slices that had valid data for all co-registered input image sets.

## Results

Table 1 shows patient characteristics. Measured lesion volumes were not significantly different between groups at any time point. NBO-treated patients had larger admission DWI lesion volumes and consequently tended to have larger absolute lesion volumes at all subsequent time points. An analysis of lesion volume change from before (baseline) to during treatment (4 h) showed significant reduction in DWI lesion size in NBO-treated patients and an increase in Control patients, consistent with previous reports [18]. At the other time points, lesion expansion was observed in both groups, but to a lesser extent in the NBO-treated group.

**Table 1:** Clinical and imaging characteristics

	Control-treated (n = 6)	NBO-treated (n = 10)
	Mean ± SD (Median)	Mean ± SD (Median)
Age (y)	71 ± 18 (71)	67 ± 16 (70)
Female	4 (67%)	5 (50%)
**Time Intervals**		
Symptom onset to admission MRI (h)	4.4 ± 2.0 (4.4)	7.3 ± 4.5 (5.9)
Admission-to-treatment MRI (h)	5.0 ± 1.4 (4.8)	4.3 ± 1.5 (3.9)
Admission-to-post-treatment MRI (h)	24.8 ± 1.9 (24.8)	24.4 ± 1.5 (24.2)
Admission-to-discharge MRI (d)	6.7 ± 1.9 (6.6)	6.0 ± 1.4 (5.8)
Acute NIHSSS	11 (9-12)	14 (9-18)
**Reperfusion**		
Admission-to-treatment	1 (17%)	0
Treatment to post-treatment	0	5 (50%)

**Measured Lesion Volume (cm^3^)**		
Admission DWI	27 ± 44 (8) cm^3^	37 ± 24 (30) cm^3^
4 h DWI	28 ± 34 (15) cm^3^	35 ± 25 (27) cm^3^
24 h DWI	32 ± 39 (15) cm^3^	49 ± 37 (43) cm^3^
Discharge	41 ± 48 (23) cm^3^	66 ± 42 (53) cm^3^

**Lesion Change (%)**		
4h*	153 ± 96 (134)%	90 ± 22 (90)%
24h	186 ± 142 (148)%	129 ± 29 (134)%
Discharge	223 ± 91 (203)%	192 ± 81 (178)%

*P = 0.036 Control-group vs NBO-group

Control- and NBO-models were trained to predict lesion development on a voxel-wise basis at 4 h, 24 h and Discharge. The coefficients for the models using all patient data in each respective cohort are shown in Table 2. These coefficients represent the relative importance of each covariate on the likelihood of lesion development. There were significant differences (P < 0.05) between the coefficients as a function of treatment type (i.e. NBO-model or Control-model) or time of lesion development (i.e. 4 h, 24 h, or Discharge). The coefficients changed over time, with the iDWI coefficient having less import for predicting outcome at later time-points for both NBO- and Control-models.

**Table 2:** GLM Coefficients for the different models (Mean ± SD) for predicting lesion development

Models	Bias	T_2_WI	ADC	iDWI	CBF	CBV	MTT	Tmax
**Models Predicting 4 h Lesion Development (during treatment)**								

Control	-11 ± 1.4§	4.0 ± 1.0§	-4.3 ± 1.3§	9.3 ± 1.2§	-0.9 ± 0.2	0.7 ± 0.1	0.5 ± 0.2	0.07 ± 0.008§

NBO	-17 ± 0.8§	2.0 ± 0.3§	0.10 ± 0.3*§	11 ± 0.5§	-0.4 ± 0.2*§	-0.02 ± 0.2*	0.9 ± 0.2	0.05 ± 0.008§

**Models Predicting 24 h Lesion Development (post-treatment)**								

Control	-3.1 ± 1.0†	8.1 ± 0.8†	-9.1 ± 1.0†	2.3 ± 0.8†	-0.7 ± 0.1	0.5 ± 0.1	0.5 ± 0.1	0.05 ± 0.007†

NBO	-13 ± 0.5†	0.6 ± 0.2†	1.0 ± 0.2†	8.9 ± 0.3†	0.2 ± 0.09†	-0.2 ± 0.08	1.3 ± 0.1	0.1 ± 0.006†

***Models Predicting Discharge Lesion Development***								

Control	-3.4 ± 0.6†	4.2 ± 0.4§	-5.1 ± 0.5§	2.7 ± 0.5†	-0.7 ± 0.1	0.5 ± 0.08	0.9 ± 0.1§†	0.06 ± 0.006

NBO	-8.8 ± 0.3†§	0.3 ± 0.1†	0.7 ± 0.2	6.1 ± 0.2†§	-0.2 ± 0.09*§	-0.06 ± 0.07*	0.9 ± 0.09§	0.1 ± 0.006†

*P > 0.05 Non-significant coefficients

†P < 0.05 vs coefficients of 4 h models, §P < 0.05 vs coefficients of 24 h models

Coefficients for Control- and NBO-models were significantly different (P < 0.05) with the exception of T_2_WI, iDWI, CBF and Tmax for 4 h and MTT for 1-week time points (displayed in light-gray).

**Figure 1:**
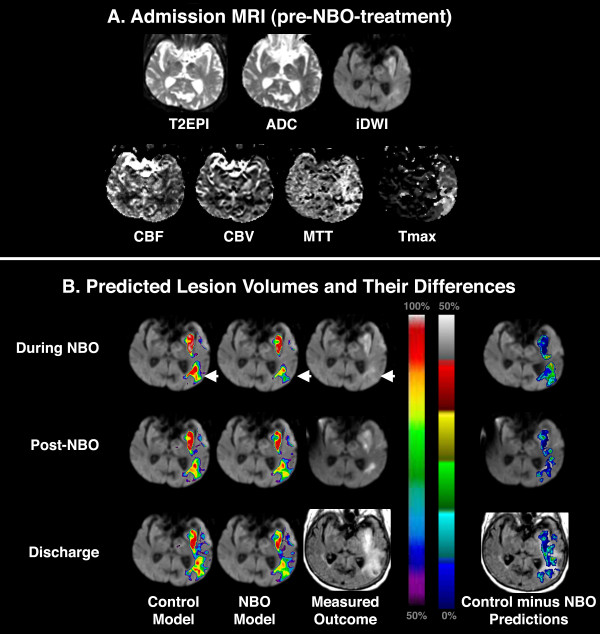
**Example 1 of Predicted Lesion Volume Development**. **(A) **Admission MRI dataset for a 79 year-old woman with stroke who was imaged 13 hours after she was last seen well and treated with NBO. **(B) **Corresponding GLM-predicted lesion risk maps (left panel) for Control and NBO-models at each subsequent time point of imaging, and overlay map (right panel) of differences between the two models showing ischemic tissue that is 'potentially salvageable' with NBO therapy. The GLM-predicted lesion volumes are asynthesis of data from the admission MRI only. In this patient, the risk of tissue infarction in DWI/PWI mismatch regions was predicted to increase over time. For clarity, only GLM-predicted lesion risk > 50% are shown overlaid on acute DWI. Note that the amounts of tissue predicted to infarct at all time-points with the Control-models were greater than their NBO-model counterparts, with difference principally in the DWI/PWI mismatch region (arrowheads). In the difference maps (B, right panel), the color scale represents infarction risk reduction as a result of NBO-therapy (conversely, larger values represent greater likelihood of infarction if the patient was given Control-treatment).

**Figure 2:**
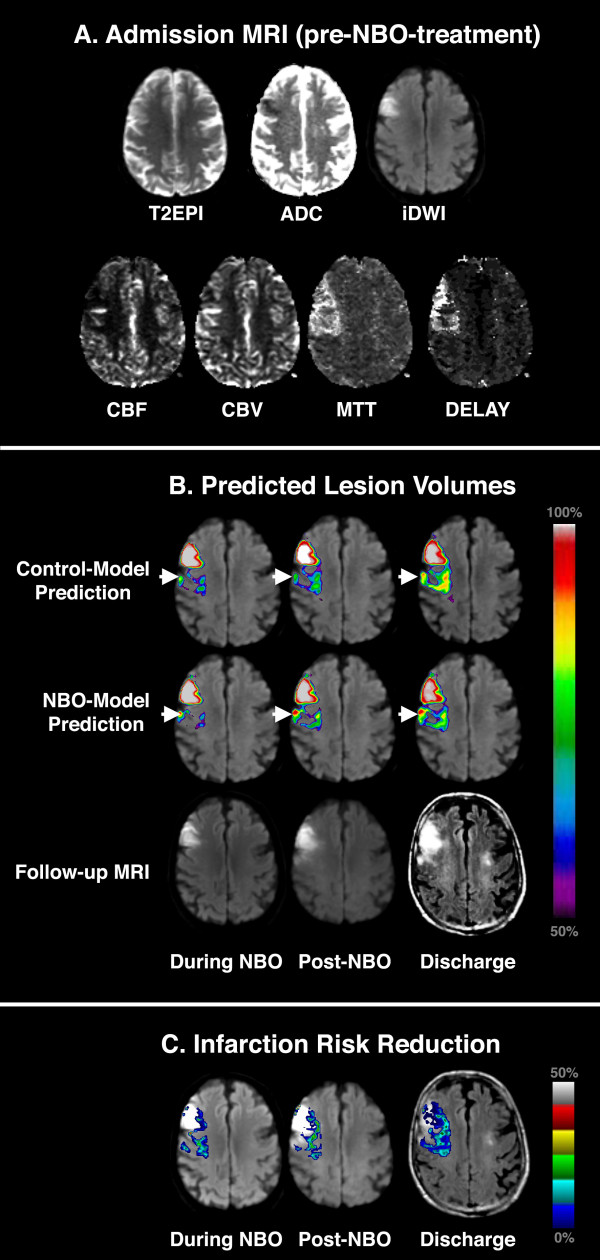
**Example 2 of Predicted Lesion Volume Development**. **(A) **Admission MRI dataset for a 64 year-old male patient imaged at 4.5 hours after stroke symptom onset who was treated with NBO. **(B) **Corresponding GLM-predicted lesion risk maps for Control and NBO-models at each subsequent time point of imaging, and **(C) **Overlay map of differences between the two models showing ischemic tissue that is 'potentially salvageable' with NBO therapy. The GLM-predicted lesion volumes are a synthesis of data from the admission MRI only. In this patient, the risk of tissue infarction in DWI/PWI mismatch regions was predicted to increase over time. For clarity, only GLM-predicted lesion risk > 50% are shown overlaid on acute DWI. Note that the amounts of tissue predicted to infarct at all time-points with the Control-models were greater than their NBO-model counterparts, with difference principally in the DWI/PWI mismatch region (arrowheads). In the difference maps (C), the color scale represents infarction risk reduction as a result of NBO-therapy (conversely, larger values represent greater likelihood of infarction if the patient was given Control-treatment).

Examples of predicted lesion development using the two models for NBO-treated patients are shown in Figures 1 and 2, along with the voxel-wise differences. Note the greatest differences between Control and NBO models in the predicted risk of lesion development are observed at 4 h.

As compared to lesion volumes predicted by NBO-models, the Control-model predicted significantly larger (P = 0.007) expected lesion volumes in the NBO-treated group at 4 h (52 ± 30 vs. 46 ± 31 cm^3^), but not at 24 h (58 ± 33 vs. 59 ± 36 cm^3^) or Discharge (74 ± 36 vs. 75 ± 38 cm^3^). The mean ± SD difference between PLV_Control _and PLV_NBO _for 4 h, 24 h, and Discharge were 5.3 ± 6.4, -1.0 ± 6.8, and -0.7 ± 5.7 cm^3^. In regions where PLV_Control _was greater than PLV_NBO_, T_2_WI, ADC, DWI, CBF, CBV and Tmax were significantly different (P < 0.05) from regions where PLV_Control _matched PLV_NBO _for 4 h predictions. For the 24 h predictions, significant differences (P < 0.05) were found for DWI, CBF, CBV, and Tmax. For the Discharge predictions, significant differences (P < 0.05) were found only for T_2_WI, DWI, CBF, CBV, and Tmax. ADC, CBF, CBV and CBF were more reduced, while T_2_WI, DWI and Tmax were higher in the matched regions where both models predicted lesion development compared to regions of discordance between the models.

**Figure 3:**
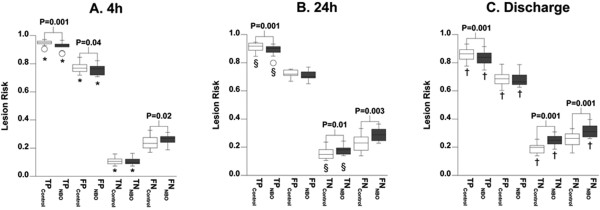
**GLM-predicted risk of lesion (mean ± SD) at (A) 4 h, (B) 24 h, and (C) Discharge in areas correctly predicted to lesion by the Control-model (TP_Control _- white box) or by the NBO-model (TP_NBO _- gray box) and in areas incorrectly predicted to lesion by the Control model (FP_Control _- white box) or the NBO-model (FP_NBO _- gray box)**. GLM-predicted lesion risk differed significantly (P < 0.05) among the 4 regions for each of the models with the exception of FN_Control _and TN_Control _at Discharge. Differences between regions over time are also shown: *P ≤ 0.01 4 h vs. 24 h. † P < 0.001 4 h vs Discharge. §P ≤ 0.001 24 h vs Discharge.

ROI analysis shows that lesion risk is greatest in TP than in FP for both models at all time points (Figure 3A-C). Lesion risk in these regions is also shown to significantly change over time.

**Figure 4:**
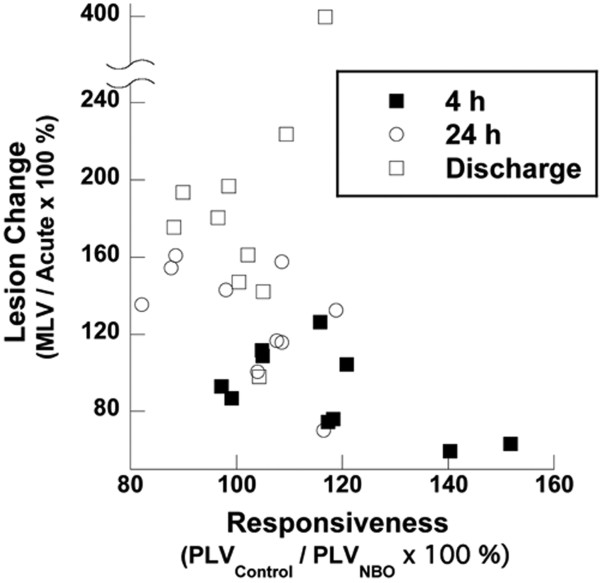
**Scatter plot of Lesion Change vs expected Responsiveness (ratio of PLV_Control _to PLV_NBO_) to therapy**. For the 4 h time point, large mismatches between the Control and NBO models were found to be associated with lesion reduction.

For all time-points, the *predicted *lesion volumes were significantly correlated with the *measured *(manually outlined) lesion volumes for both the Control-models (4 h: R = 0.94 P < 0.0001; 24 h: R = 0.95 P < 0.0001; Discharge: R = 0.88 P < 0.001) and the NBO-treatment models (4 h: R = 0.97 P < 0.0001; 24 h: R = 0.97 P < 0.0001; Discharge: R = 0.83 P < 0.01). Lesion Change at 4 h had a tendency to be correlated with expected responsiveness to therapy, i.e. the ratio of PLV_Control_/PLV_NBO_, (R = -0.61 P = 0.06), but was poorly correlated at 24 h (R = -0.56 P = 0.09) and at Discharge (R = 0.52 P = 0.12) (Figure 4). When DWI lesions were observed to shrink rather than expand at the 4 h time point, PLV_Control_/PLV_NBO _was 121 ± 22%.

## Discussion

In this study, we used objective methods to validate prior observations showing the safety and efficacy of 8-hour NBO in selected patients with acute ischemic stroke. Further, we show that paired analysis of expected outcomes under two therapeutic settings (control and NBO) can be used to evaluate effects of novel therapeutic interventions. Using multivariate voxel-based algorithms, we can compare the expected outcome of each voxel of tissue (were the natural cascade of ischemic events to proceed unimpeded), versus their fate with therapy. Despite the imbalanced number of subjects or data in small pilot studies that occurs regardless of randomization, as was true for this investigation and reflected by the slightly larger DWI lesions in the NBO-arm, we were still able to detect an alteration in the patient's lesion evolution from NBO-therapy.

In our NBO pilot study [18], oxygen therapy transiently improved clinical deficits and DWI lesion volumes in stroke patients presenting with mismatches in lesion volumes on DWI and PWI. This transient beneficial effect of NBO has also been shown in rodent stroke studies [10-17]. Similarly in this study we found that DWI lesion volume growth with respect to baseline in the NBO-arm was reduced at 4 h compared to the Control-arm. The current study extends these change-from-baseline volumetric methods in individual modalities since combining multiple imaging modalities have been shown to more accurately predict tissue infarction than any currently existing individual technique [1,2]. Predictive modeling approaches also have the potential to test whether a new therapy worsens tissue outcome. A theoretical concern with oxygen therapy is an increased risk of generating toxic oxygen free radicals [31]. By showing that the predicted lesion volumes assuming NBO-treatment was not greater than those assuming room-air-treatment, our objective data provides evidence that NBO-therapy may not worsen tissue outcome at Discharge.

In this study, we extend our previous GLM modeling approach [1,7], which were limited to "natural history models," by developing tissue-signature models that use pre-treatment data to predict expected tissue outcome if a novel therapy was to be given. Our results showed that during therapy FP_Control _was significantly higher than FP_NBO_, suggesting NBO-treatment lowered the risk of lesion development in these regions at 4 h. This effect was not noted at the other time points. Further, we found that greater mismatch between predicted outcome using "natural history models" versus "NBO-treatment model" was associated with smaller lesion expansion and in some cases lesion reduction, suggesting these models may be useful in selecting patients most likely to benefit from NBO therapy. We speculate that risk maps that synthesize multiparametric imaging, such as those presented here and by others [2,32], may provide an objective quantitative means for *a priori *predicting tissue outcome on an individual voxel basis before therapy administration. The algorithms proposed in this study are thus initial steps towards developing tools that will aid the clinician to tailor therapy on an individual patient basis since these models can theoretically be used to determine whether one therapy, e.g. NBO or rt-PA, will produce smaller lesion volumes as compared to another therapy. This may be useful for neuroradiologists and stroke physicians who are faced with an increasing myriad of imaging modalities [33] aimed at interrogating the state of the ischemic tissue.

Insight into the mechanisms of tissue salvage by these interventions may potentially be gleaned by comparing the coefficients for the different models. For example, we found that MTT plays a greater role in the NBO-models than in Control-models in predicting which tissue is likely to develop into a lesion at each of the time-points. ADC and CBV appear to matter less in the NBO during-treatment 4 h models, suggesting that with NBO, the presence in a voxel of low ADC and low CBV are less likely to result in lesion. This suggests that there may be brief neuroprotection bestowed on at-risk tissue by NBO despite lack of reperfusion (Table 1). In addition, we note that iDWI progressively assumes less weight with Tmax playing a more important role over time for the NBO-model and less importance for the Control-model. We theorize that this is due to preservation of the DWI/PWI mismatch in the early stages of lesion development by NBO therapy, with infarct expansion into the area of abnormality delineated by Tmax maps, which have been speculated to be a reflection of the extent of collateral flow and hence tissue salvage [23,34,35]. We also observed greater variability in NBO-models than in the Control-models. We speculate that this is in part due to greater heterogeneity in patient response to NBO therapy, which are likely mediated by several clinical factors that cannot be captured in neuroimaging, such as age, gender, blood pressure, blood glucose levels, or hematocrit, which have been shown to effect rates of recanalization and hemorrhagic transformation after intravenous or intra-arterial thrombolysis [36]. Although randomization will somewhat compensate for these differences, it may be necessary to explicitly incorporate non-imaging covariates in MRI-based multivariate algorithms in order to improve prediction of tissue outcome in patients given novel intervention.

Tissue-based models have some limitations. For example, the accuracy of predictions depends on the training data [7]. For these models to be applicable to a general stroke population, one would need both larger training data sets and more sophisticated models that incorporate factors that mediate lesion evolution. The imbalance in the rate of reperfusion in our Control group (17%) compared to NBO group (50%) could partly explain the differences observed between the model performances. We have shown previously that models predicting outcome of patients after thrombolysis who experience early reperfusion perform poorly, probably due to salvage of tissue that would have otherwise infarcted [7]. Likewise, NBO-therapy may play an indirect role in reperfusion, as evidenced by reports of increased cerebral blood flow and blood volume during NBO in peri-infarct regions [15-17]. However, future studies predicting outcome in dichotomized analyses between patients who reperfuse and those who do not in both groups should be performed to better discriminate the effects of reperfusion on NBO-treated patients. Another important issue before these models can be used in a clinical decision-making setting is reproducibility of model parameters across centers. For example, the coefficients for the Discharge Control-model may differ significantly between studies [7] due to factors such as different onset-to-MRI times, patient inclusion criteria, patient populations, and PWI techniques.

## Conclusion

Our findings indicate that predictive algorithms can be used to estimate risk of lesion development for patients given therapeutic interventions such as NBO. Differences between predicted results for control models and treatment models were found on a volumetric basis (i.e. predicted lesion volume) as well as on a voxel-wise basis (i.e. probability of lesion development) suggesting a therapeutic benefit for NBO at 4 h. Further studies are needed to determine the utility of MRI predictive algorithms in stroke clinical trials, and to confirm the safety and efficacy of NBO in acute ischemic stroke.

## Abbreviations

NBO: Normobaric oxygen therapy; rt-PA: Recombinant tissue plasminogen activator; MRI: Magnetic resonance imaging; DWI: Diffusion-weighted imaging; PWI: Perfusion-weighted imaging; MRA: Magnetic resonance angiography; CBF: Cerebral blood flow; CBV: Cerebral blood volume; MTT: Mean transit time; Tmax: Time to maximum delay; ADC: Apparent diffusion coefficient; T2WI: T2-weighted image; MLV: Measured lesion volume; PLV: Predicted lesion volume; ROI: Region of interest; TP: True positive; FP: False positive; TN: True negative; FN: False negative.

## Competing interests

O.W. and A.G.S are co-inventors on US Patent 7,020,578, "Method for evaluating novel, stroke treatments using a tissue risk map." The other authors declare that they have no competing interests.

## Authors' contributions

Study concept and design: OW, AGS, ABS; Acquisition of data: ABS, TB, LR, MZ, PWS, OW, AGS; Statistical analysis: OW; Analysis and interpretation of data: OW, AGS, ABS; Drafting of the manuscript and critical revisions: OW, AGS, ABS. All authors read and approved the final manuscript.
